# Supplementary material for: Hearing and cognitive decline in aging differentially impact neural tracking of context-supported versus random speech across linguistic timescales
Source: PLoS One. 2024 Dec 6;19(12):e0313854. doi: 10.1371/journal.pone.0313854 (PMC11623803; doi:10.1371/journal.pone.0313854)
Supplement: Attachment — Submitted filename: Response letter to reviewers.pdf [file pone.0313854.s001.pdf]

Universität Zürich[UZH]

Department of Computational Linguistics

University of Zurich
Department of Computational Linguistics
Computational Neuroscience of Speech and Hearing
Andreasstrasse 15
8050 Zurich, Switzerland
cl.uzh.ch/neuro

UZH, CL

PLOS One

Elena Bolt
Researcher
+41 44 634 62 44
elena.bolt@uzh.ch

Zurich, October 25, 2024

Reply to the reviewers' comments on our manuscript submitted to PLOS One

Dear Reviewers and Dr. Rothermich,

We would like to express our sincere gratitude for the opportunity to enhance our manuscript through your valuable feedback. We have carefully considered all of your comments, which have led to minor revisions.

We appreciate the reviewers' insightful input. We are particularly grateful to Reviewer 1 for their friendly and positive feedback, as well as to Reviewer 2, Dr. Kries, for providing constructive comments that have significantly improved our manuscript.

This letter is organized into two sections: a point-by-point response to Reviewer 1, followed by a point-by-point response to Reviewer 2. We hope that you find our revisions satisfactory and that our manuscript merits further consideration.

Best regards,

Elena Bolt and Nathalie Giroud

Reply to Reviewer 1

We'd like to express our sincere thanks to the anonymous reviewer for their kind words and positive feedback on our manuscript. We're really pleased that they found the work both interesting and well-written.

Regarding their comments: the missing references were an oversight on our part, and we've now corrected them. We appreciate the reviewer bringing this to our attention. As for the figure and table placement, we understand the confusion. Unfortunately, the journal's LaTeX template limits our control over exact positioning during submission. While we followed the guideline to place figures and tables after the first paragraph where they're cited, the compiled version doesn't always display them perfectly. We'll ensure this is addressed in the final version once the manuscript is accepted.

We again thank the reviewer for their time and valuable insights.

Reply to Reviewer 2

We sincerely thank Dr. Kries for her positive review of our paper and her valuable feedback and her time.

We appreciate the reviewer for bringing the missing references to our attention. This was a technical error that occurred shortly before uploading the document, and we have since corrected it throughout the manuscript. Below, we will address each of her points in detail. For reference, we will indicate the line numbers in the PDF file with tracked changes (LaTeX difference file). However, for the sake of readability, we have included the relevant text from the clean revised manuscript in this letter.

---

line 51-53: "our previous study" comes as a surprise to the reader, reformulate

We have made the following rewording:

> "To build on these findings, we conducted a follow-up study using natural continuous speech instead of syllables, aiming to elicit subcortical and cortical auditory responses. However, the follow-up study did not confirm altered encoding in cognitive decline [21]." (L51–54)

We hope that the new wording provides more clarity. We thank the reviewer for pointing this out to us.

---

line 85: "discrete chunks of acoustic units", shouldn't it rather be linguistic units?
line 88: add number of participants
line 89: explain what matrix sentences are, not everyone knows that

The reviewer is right, so we have reworded the description from "acoustic" to "linguistic" units (L88). Furthermore, we have added the number of participants (L91). And finally, we inserted an explanation of matrix-style sentences on L92.

- *line 85: "discrete chunks of acoustic units", shouldn't it rather be linguistic units?*
- *line 88: add number of participants*
- *line 89: explain what matrix sentences are, not everyone knows that*

The reviewer is right, so we have rephrased the description from "acoustic" to "linguistic" units (L88).

Furthermore, we have added the number of participants and inserted an explanation of matrix-style sentences on L92:

> "In this study, 45 participants aged 60 to 83 years listened to two sets of in-house--designed, matrix-style sentences (i.e., sentences with a fixed grammatical structure) that either provided supportive sentence context or were random, while we recorded their electroencephalography (EEG)." (L91–92)

We hope that this improves the clarity of the introduction and thank the reviewer for her comment.

line 134-136: change order of sentences

We changed the order of the sentences as suggested by the reviewer:

> "Participants 135 performed well, answering correctly in 96 ± 4% of the trials. No participant scored below 136 85% correct responses. The task was designed to be easy, and we excluded all trials with 137 incorrect responses from neurophysiological analyses." (L140–142)

The text now makes more sense. Thank you very much.

---

line 160: can you add a plot to the supplementary information with the AEPs of each subject individually laid over each other (averaged across the relevant EEG channels)

We appreciate the reviewer's interest in exploring the AEPs in more detail. The figure below shows the 45 individual AEPs overlaid, with the overall average (black line) estimated using the frontocentral electrodes (F3, FC1, FC5, FC6, FC2, and F4), as detailed in the manuscript.

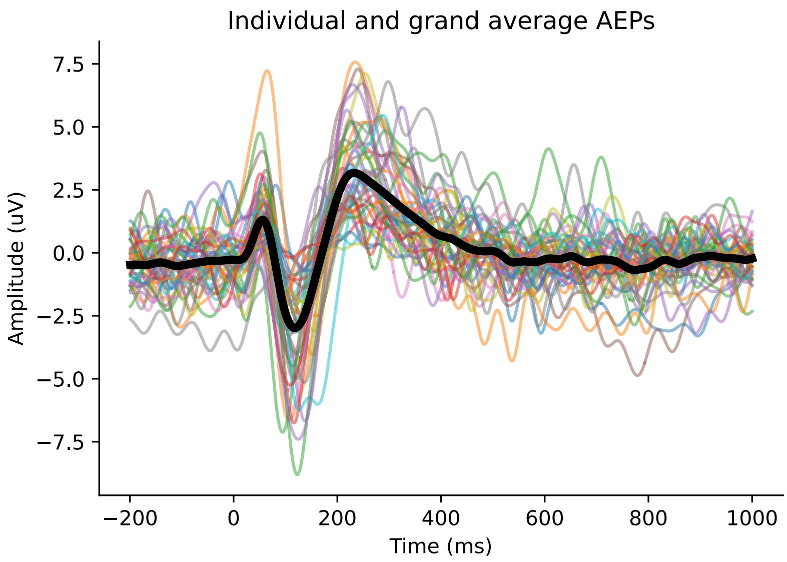

While we understand the curiosity behind this request, we believe that this plot doesn't provide much additional insight compared to Figure 2A. For this reason, we would prefer not to include it in the supplementary information. However, we are happy to provide it here for the sake of transparency and clarity.

- *line 209-211: can you elaborate why you assume that why the parietal cluster result would originate from frontocentral electrodes? And the formulation is a bit cryptic, i.e., "Across the scalp" to me means all electrodes, but then you talk about the parietal cluster. Also I think the idea of also including clusters in your statistical analysis is not explained properly before you report the results, so please do that.*
- *line 225-227: idem previous comment*
- *line 710-711: "ensuring" is a bit too strong, replace with "targeting", or "trying to target"*

We apologize for the formulation, which was indeed confusing. In the linear mixed model on the word rate, the parietal cluster was significant, with a negative Beta estimate, which indicates that in this cluster, the PLV was significantly lower compared to the frontal reference cluster. In the revised manuscript, we clarified:

> "We found a main effect for the parietal cluster, which showed a significant decrease in tracking at the word rate, indicating that speech tracking was mainly driven by frontocentral electrodes (Figure 3A)." (L217–220)

Similarly, we adapted this for tracking at the syllable rate:

> "The parietal cluster showed a significant decrease in tracking at the syllable rate, again indicating that this speech tracking was mainly driven by frontocentral electrodes (Figure 3A)." (L250–251)

Furthermore, we added the following explanation to the introduction:

> "In the statistical analyses, we included three electrode clusters, a frontal (F), central (C), and parietal (P) cluster, to account for the topographical distribution of the tracking values." (L199–201)

Please also note that we have included a subsection on "electrode clusters" explaining the rationale for this approach. In this section, we have also included the reviewer's suggestion to replace "ensuring" with "targeting":

> "Following an approach we adopted in a previous study [22], we preselected nine midline electrodes for detailed analysis. These electrodes—F3, Fz, F4, C3, Cz, C4, P3, Pz, and P4—were grouped into three regional clusters: frontal (F), central (C), and parietal 745 (P). This method enabled us to incorporate the activity of individual electrodes in our analysis while also conducting hierarchical modeling at the cluster level. The chosen electrodes were strategically positioned near the auditory cortex, targeting their relevance to neural speech processing. By covering a broad scalp area, we avoided making prior assumptions about the exact location of neural generators." (L742–750)

We thank the reviewer for pointing all this out to us and are convinced that with her input the revised text should now be clearer.

Figure 3B: add individual data points please, you can make them transparent if you think the plot becomes too busy

In Figure 3B, we aimed to illustrate the main effect of PTA on PLV. Since this analysis is based on nested data, it includes data points from all participants, clusters, and items. Below is the plot with individual data points added.

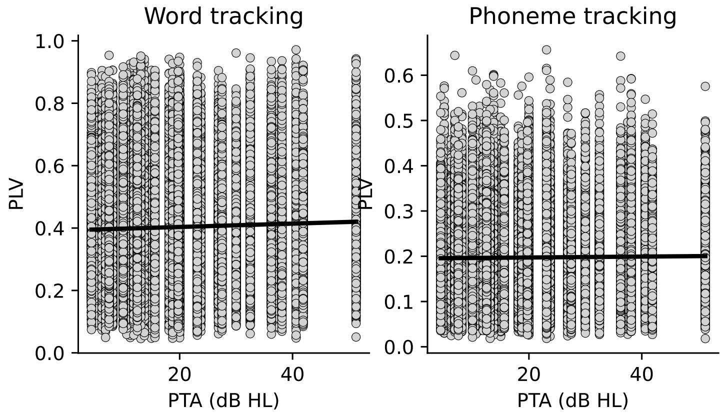

We appreciate the suggestion to use transparency to reduce visual clutter. However, even with transparency, the plot remains very busy and difficult to interpret. This is why we chose to display only the regression line, its slope, and the relevant PLV range in the original figure. Including individual data points, even transparent ones, would still detract from the figure's clarity. For these reasons, we would prefer to keep the current simplified version.

---

line 230-232: are the post hoc comparisons not significant (even though the interaction is) due to correction for multiple comparisons? I am just trying to understand how come none of the post hoc comparisons was significant.

We thank the reviewer for this important question. Yes, the post-hoc pairwise comparisons were not significant due to correction for multiple comparisons using the Tukey method (included in the *emmeans*-package).

While the interaction between MoCA group and condition was significant, the post-hoc tests, which examine specific pairwise differences within each condition, did not reach significance after the correction. This discrepancy arises because the interaction term tests whether the relationship between the factors differs across levels, while the post-hoc tests focus on individual comparisons. The Tukey correction likely reduced the statistical power, potentially masking smaller effects that contributed to the significant interaction. In other words, the overall interaction was significant, but the specific differences within each condition were not large enough to remain significant after correction.

line 259-261: “As hearing ability increased, tracking at the phoneme rate increased.” this is the opposite of what the plot 3B shows, isn’t it? You measure PTA in dbHL, that means that the higher the PTA value is, the worse their hearing ability is. Thus, it should be “As hearing ability decreased, tracking at the phoneme rate increased.” Make sure throughout the paper that you get this correct.

Many thanks to the reviewer for her careful review and for pointing out this mistake. We have ensured consistency in our terminology throughout the revised manuscript. As stated on L 243 and L286, we now correctly describe the relationship between hearing ability and tracking.

---

line 267-268: “a lower chance of correct responses”, this seems to be a bit of a complicated formulation to say that they had decreased response accuracy. Can you change that throughout the manuscript please?

We have revised the phrasing as suggested, changing “a lower chance of correct responses” to “decreased response accuracy” in L26, L293, and L481. We agree that this sounds much better. Thank you very much.

---

line 290-293: maybe add already here that the absence of an effect of cognitive decline on N400 might be related to your low MoCA group being very mild, with 9 participants actually scoring only one point below the clinical cut-off threshold

We thought this was a very good suggestion from the reviewer and added the explanation:

> “One potential explanation for the lack of significant findings in the N400-like component could be the mild cognitive decline in the low MoCA group, with nine participants scoring only one point below the clinical cut-off threshold.” (L320–323)

---

line 341-344 and line 361-364: these speculations about non-significant effects are a bit too speculative, I would remove that

We agreed with the reviewer and removed the paragraph completely (L391–393). Thank you very much.

---

section Strengths and limitations of the study: add that you have a limited variability of hearing loss in your sample, which might veil effects of PTA

We thought this was good advice from the reviewer and have therefore added a paragraph to the Strengths and limitations:

> “**Limited hearing loss gradients reduce generalizability**. Our sample had relatively good hearing for their age, resulting in low variability in hearing loss. While this minimized confounding factors, it may have concealed the effects of hearing loss on speech tracking measures. Consequently, we didn't find consistent significant effects across all measures. Including participants with a broader range of hearing abilities in future studies could provide a clearer understanding of how cognitive decline interacts with hearing loss in speech processing and enhance the generalizability of the findings.” (L–422–229)

We again thank Dr. Kries for her insightful comments and the time she dedicated to our manuscript.
